# Hematological Parameters and Procalcitonin as Discriminants between Bacterial Pneumonia-Induced Sepsis and Viral Sepsis Secondary to COVID-19: A Retrospective Single-Center Analysis

**DOI:** 10.3390/ijms24065146

**Published:** 2023-03-07

**Authors:** Emanuel Moisa, Madalina Dutu, Dan Corneci, Ioana Marina Grintescu, Silvius Negoita

**Affiliations:** 1 Department of Anaesthesia and Intensive Care Medicine, Faculty of Medicine, ‘Carol Davila’ University of Medicine and Pharmacy, 020021 Bucharest, Romania; 2 Clinic of Anaesthesia and Intensive Care Medicine, Elias University Emergency Hospital, 011461 Bucharest, Romania; 3 Clinic of Anaesthesia and Intensive Care Medicine, Dr. Carol Davila Central Military Emergency University Hospital, 010825 Bucharest, Romania; 4 Clinic of Anaesthesia and Intensive Care Medicine, Clinical Emergency Hospital of Bucharest, 014461 Bucharest, Romania

**Keywords:** sepsis, viral sepsis, procalcitonin, RDW, COVID-19, leukocytes, monocytes, PLR, NLR

## Abstract

Bacterial and viral sepsis induce alterations of all hematological parameters and procalcitonin is used as a biomarker of infection and disease severity. Our aim was to study the hematological patterns associated with pulmonary sepsis triggered by bacteria and Severe Acute Respiratory Syndrome–Coronavirus–type-2 (SARS-CoV-2) and to identify the discriminants between them. We performed a retrospective, observational study including 124 patients with bacterial sepsis and 138 patients with viral sepsis. Discriminative ability of hematological parameters and procalcitonin between sepsis types was tested using receiver operating characteristic (ROC) analysis. Sensitivity (Sn%), specificity (Sp%), positive and negative likelihood ratios were calculated for the identified cut-off values. Patients with bacterial sepsis were older than patients with viral sepsis (*p* < 0.001), with no differences regarding gender. Subsequently to ROC analysis, procalcitonin had excellent discriminative ability for bacterial sepsis diagnosis with an area under the curve (AUC) of 0.92 (cut-off value of >1.49 ng/mL; Sn = 76.6%, Sp = 94.2%), followed by RDW% with an AUC = 0.87 (cut-off value >14.8%; Sn = 80.7%, Sp = 85.5%). Leukocytes, monocytes and neutrophils had good discriminative ability with AUCs between 0.76–0.78 (*p* < 0.001), while other hematological parameters had fair or no discriminative ability. Lastly, procalcitonin value was strongly correlated with disease severity in both types of sepsis (*p* < 0.001). Procalcitonin and RDW% had the best discriminative ability between bacterial and viral sepsis, followed by leukocytes, monocytes and neutrophils. Procalcitonin is a marker of disease severity regardless of sepsis type.

## 1. Introduction

Sepsis is defined as a life-threatening condition associated with variable degrees of organ dysfunction in a host with an aberrant response to infection [1]. Although bacteria, viruses and parasites can be the causative agent of sepsis, in clinical practice bacterial sepsis prevails [2,3]. The Coronavirus Infectious Disease-19 (COVID-19) pandemic had a major impact on how we approach patients with viral sepsis, and was also an opportunity to deepen our understanding regarding its mechanisms.

Whether bacterial or viral, sepsis is associated with profound disturbances of the normal homeostasis secondary to increased production of pro-inflammatory cytokines–the so called cytokine storm in COVID-19 sepsis, while in bacterial sepsis is known as the systemic inflammatory response syndrome (SIRS) [1,4]. The use of SIRS in the diagnosis of sepsis remains a matter of debate, as SIRS is present in various conditions [1,5].

Bacterial and viral sepsis are associated with qualitative and quantitative disturbances of all hematological parameters [2,4,5,6]. Quantitative changes of leukocytes or immature forms are part of the SIRS criteria [7]. Bacterial infections are associated with both leukocytosis and neutrophilia, but also with a high production of the immature granulocytes [2,5,8]. The same observation was made for COVID-19 [6,9]. Song et al. [10] studied the hematological differences between COVID-19 and bacterial pneumonia. Patients with bacterial pneumonia presented a significantly higher frequency for leukocytosis (*p* = 0.011), neutrophilia (*p* = 0.021) and monocytosis (*p* = 0.023). No difference was observed regarding neutrophil-to-lymphocyte ratio (NLR) and lymphocyte-to-monocyte ratio (LMR) (*p* > 0.05). Leukocytes were good discriminants between bacterial pneumonia and COVID-19 (area under the curve = 0.778) [10].

Regarding lymphocytes, in severe and critical forms of COVID-19, their number is decreased and was associated with disease severity and progression, but also with mortality [6,11,12]. In these patients, A CD8 T cell number of less than 75/μL is strongly associated with mortality (odds ratio = 3.982, 1.132–14.006, *p* < 0.001) [11]. In bacterial sepsis, persistent lymphopenia is associated with immune paralysis and in patients with suspected infection can differentiate between septic and non-septic patients [4,12]. Monocytes count and function alterations are frequently observed in patients with bacterial sepsis [5,13], while in COVID-19, monopenia and abnormal monocytic activity prevails [14]. Moreover, monocyte distribution width (MDW) has good discriminative ability for the diagnosis of bacterial sepsis (area under the curve = 0.727). A MDW value >20.0 has good accuracy for sepsis diagnosis [15]. A MDW value >24.685 U is an indicator of progression to viral sepsis and poor prognosis in patients with COVID-19 [16].

Moser et al., studied the immune response in patients with COVID-19 ARDS and patients with bacterial sepsis secondary to pneumonia or other causes. The authors found that, during the first week, leukocytes have a reduced ability to produce IL-2 (interleukin-2), TNF-α (tumor necrosis factor-α) and IFN-γ (interferon-γ) in response to a non-self in patients with bacterial sepsis. In COVID-19 patients, leukocytes were still able to produce these cytokines. This could explain the different evolution of these two groups suggesting that: (i) patients with bacterial sepsis present with a higher degree of immune paralysis, (ii) in COVID-19 sepsis, a continuous, hyperinflammatory state is more probable during the first 7 days [4].

Ito et al., found that transcripts related to mitochondria were upregulated in COVID-19, while, those related to neutrophils activation were upregulated in bacterial sepsis. Overall, transcripts related to neutrophils were upregulated in both types of infection compared to healthy controls [17]. Their observations are similar with those of other authors who found that the peripheral count of immature neutrophils and their level of activity is related to COVID-19 severity [9].

Derived hematological indices, such as NLR, monocyte-to-lymphocyte ratio (MLR), platelet-to-lymphocyte ratio (PLR), derived neutrophil-to-lymphocyte ratio (dNLR) and systemic inflammatory index (SII) were studied in COVID-19 and bacterial sepsis and as factors distinguishing between viral and bacterial pneumonia in critically-ill patients [6,18,19]. NLR, although not specific, was proposed as a marker of diagnosis in sepsis. and also, as an instrument that can differentiate between sepsis and septic shock [20]. Septic shock can be excluded by a NLR < 3 and should be considered in patients with NLR values between 10 and 15. In patients with NLR > 15 septic shock is highly probable. Lastly, a NLR value between 3 and 10 represents a grey zone with no diagnostic power and patients should be treated independently of NLR value [20]. In COVID-19, a NLR value >6.11 was proposed as a diagnostic and triage tool for diagnosis and choice of therapy [21]. Results regarding the discriminative ability of procalcitonin between bacterial and viral pneumonia are conflicting and some authors suggest that increased levels of procalcitonin are highly associated with the degree of organ involvement and one should cautiously use its value when diagnosing and treating a suspected bacterial infection [22,23,24].

Many sepsis subtypes are described based on advanced laboratory tests, but the actual clinical practice is still far from using them on a daily basis [1,2,5,25]. Thus, our aim was to study the effects of bacterial and viral sepsis on the hematological parameters routinely measured in critically-ill patients (absolute count of blood cells, derived hematological indices) and procalcitonin level and describe the main differences. Another aim of this research was to study the discriminative ability of the aforementioned parameters.

## 2. Results

### 2.1. Baseline Characteristics of the Study Population

**Figure 1:**
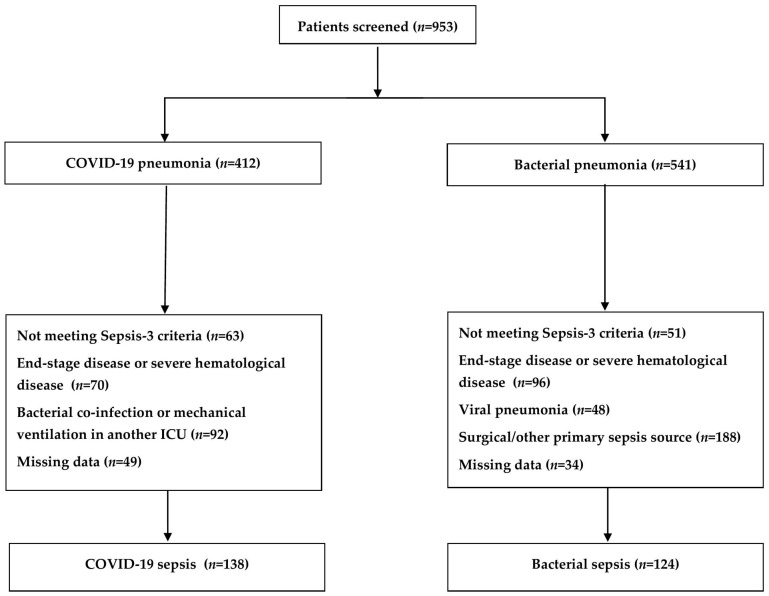
Flowchart of the study population.

A total of 262 patients met the inclusion criteria (Figure 1): 124 with bacterial sepsis and 136 with COVID-19 sepsis. Median age for the whole sample was 68 years [IQR: 58–77] and was significantly different between the two groups, patients with bacterial sepsis being older (*p* < 0.001). Out of 124 patients with bacterial sepsis, 74 were male, while in the COVID-19 sepsis group 84 were male with no difference between groups (*p* = 0.844). Regarding associated diseases, chronic kidney disease had a significantly higher frequency among patients with bacterial sepsis (*p* < 0.001), while the frequency for obesity, diabetes mellitus, cardiac, respiratory or hepatic disease did not differ between groups (*p* > 0.05). Overall, patients with bacterial sepsis had significantly higher Charlson Comorbidity Index values (*p* < 0.001) (Table 1).

Lower P/F ratio values were observed in patients with COVID-19 sepsis (*p* < 0.001), but mechanical ventilation need was similar between groups (74.6% vs. 71.8%, *p* = 0.601). On the other hand, vasopressor support was needed more often in bacterial sepsis (*p* < 0.001), patients from this group having higher SOFA score values (*p* < 0.001) (Table 1). Lastly, among patients with bacterial sepsis, pneumonia was clasiffied as following: 60 (48.4%) presented with community-acquired pneumonia, 34 (27.4%) had healthcare-associated pneumonia and 30 (24.2%) had hospital-acquired pneumonia.

### 2.2. Hematological Parameters and Procalcitonin Analysis at the Moment of Diagnosis

Higher than normal values with significant differences between bacterial and COVID-19 sepsis patients were observed for the median values of white blood cells (*p* < 0.001), neutrophils (*p* < 0.001), immature granulocytes (*p* = 0.023), eosinophils (*p* < 0.001) and basophils (*p* < 0.001). Moreover, patients with COVID-19 sepsis presented significantly lower values for lymphocytes (*p* = 0.005) and monocytes (*p* < 0.001). Lower platelets values were observed in patients with bacterial sepsis (*p* < 0.001) with no difference between subjects regarding the PDW values (*p* = 0.215). Finally, in bacterial sepsis, increased RDW values (15.7% [IQR: 15–17.2] vs. 13.6% [IQR: 12.8–14.5], *p* < 0.001) and decreased hemoglobin levels (*p* < 0.001) were noted (Table 1).

Regarding the derived hematological indices, no differences were observed between bacterial and COVID-19 sepsis for dNLR (*p* = 0.584) and SII (*p* = 0.314), while PLR values were significantly higher in the latter group (*p* < 0.001). Furthermore, higher NLR (*p* = 0.01) and MLR (*p* < 0.001) median values were noted when comparing bacterial with COVID-19 sepsis.

### 2.3. Discriminative Ability of the Hematological Parameters and Procalcitonin between Bacterial and Viral Sepsis

**Figure 2:**
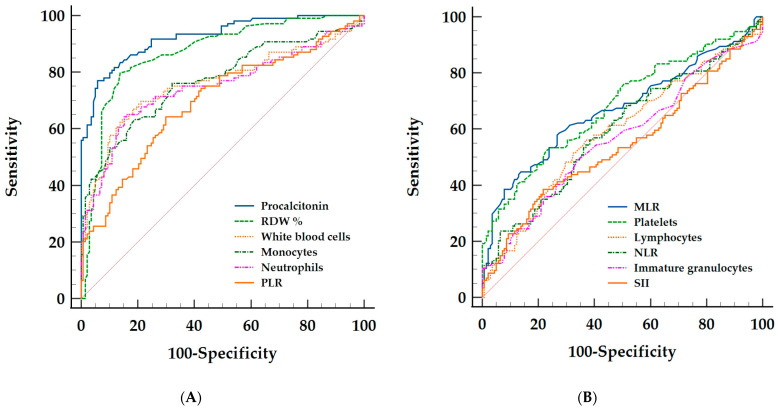
Discriminative analysis for the diagnosis of bacterial versus viral sepsis of procalcitonin and hematological parameters. (**A**) Parameters with an area under the curve >0.7; (**B**) Parameters with an area under the curve <0.7. Bacterial sepsis group: 124 patients, COVID-19 sepsis group: 138 patients. RDW% = red blood cell distribution width; PLR = platelet-to-lymphocyte ratio; MLR = monocyte-to-lymphocyte ratio; NLR = neutrophil-to-lymphocyte ratio; SII = systemic inflammatory index.

ROC curves were plotted in order to study the discriminative ability of the hematological parameters and procalcitonin between bacterial and viral sepsis. Among the hematological parameters (Table 2), RDW% had a very good discriminative power with an area under the curve (AUC) of 0.87 (95% CI: 0.82–0.91, *p* < 0.001) (Figure 2A). A RDW% cut-off value of > 14.8 (95% CI: 14.6–15.2) had a Sn of 80.7% (95% CI: 72.6–87.2) and a Sp of 85.5% (95% CI: 78.5–90.9) with a +LR of 5.56 (95% CI: 3.68–8.42) and a –LR of 0.23 (95% CI: 0.16–0.33) for the diagnosis of bacterial sepsis. No other hematological parameter had an AUC value higher than 0.8. The ROC curves plotted for white blood cells, monocytes, neutrophils and eosinophils performed in a similar manner (Figure 2A), having good discriminative ability, with AUCs between 0.72 and 0.78 (*p* < 0.001). No significant difference between AUCs values were observed (*p* > 0.05). The identified cut–off values for the diagnosis of bacterial sepsis together with Sn, Sp, +LR and –LR are found in Table 2.

Out of all hematological derived indices, PLR had good discriminative ability, with an AUC of 0.71 (95% CI: 0.64–0.76, *p* < 0.001). MLR and NLR had a fair discriminative ability (*p* < 0.05) (Figure 2A). The model was rejected for SII and dNLR (*p* > 0.05) (Figure 2B).

Lastly, procalcitonin had the best discriminative ability among all the measured parameters with an AUC of 0.92 (95% CI: 0.87–0.95), the model having excellent predictive power (Figure 2A). The identified cut-off value using the Youden index was of >1.49 ng/mL (95% CI: 1.28–1.9). For this cut-off value, the test has a Sn of 76.6% (95% CI: 68.2–83.7), a Sp of 94.2% (88.9–97.5), a +LR of 13.22 (95% CI: 6.7–21.1) and –LR of 0.25 (95% CI: 0.18–0.35) when used in the diagnosis of bacterial sepsis. No difference between procalcitonin and RDW% AUCs were observed (*p* = 0.12), while their AUCs were significantly higher than all other AUCs plotted (*p* < 0.001). The complete ROC analysis is summarized in Table 2.

### 2.4. Correlations between SOFA Score and Hematological Parameters and Procalcitonin as Markers of Disease Severity

**Figure 3:**
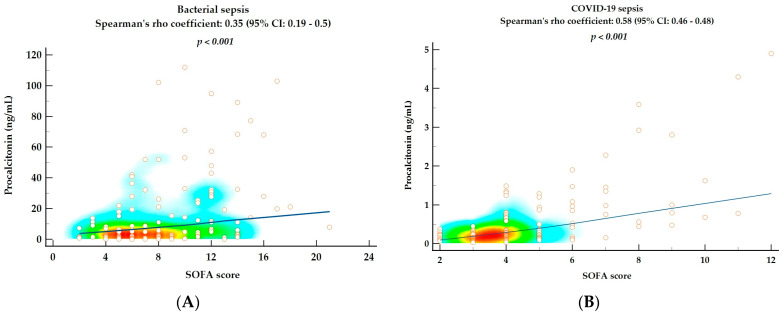
Correlation analysis between procalcitonin and SOFA (Sequential Organ Failure Assessment) score using Spearman’s rho coefficient. (**A**) Scatter diagram with heat map and trend line for the correlation between procalcitonin and SOFA score in bacterial sepsis; (**B**) Scatter diagram with heat map and trend line for the correlation between procalcitonin and SOFA score in COVID-19 sepsis. A LOESS smoothing span of 99% was used for the trend lines.

Bivariate analysis using Spearman’s rho correlation coefficient in patients with bacterial sepsis revealed significant and positive correlations between SOFA score and RDW% value (Spearman’ rho coefficient = 0.25 (95% CI: 0.08–0.41), *p* = 0.006) and between SOFA score and procalcitonin (Spearman’ rho coefficient = 0.35 (95% CI: 0.19–0.5), *p* < 0.001) (Figure 3A). Thus, higher RDW% and higher procalcitonin values are associated with higher degrees of disease severity.

On the other hand, in patients with COVID-19 sepsis, the following positive correlations between SOFA score and hematological parameters were found: NLR (Spearman’ rho coefficient = 0.27 (95% CI: 0.11–0.42), *p* = 0.001), dNLR (Spearman’ rho coefficient = 0.22 (95% CI: 0.05–0.37), *p* = 0.011), neutrophils (Spearman’ rho coefficient = 0.26 (95% CI: 0.09–0.41), *p* = 0.002) and white blood cells (Spearman’ rho coefficient = 0.23 (95% CI: 0.06–0.38), *p* = 0.008). Similar with bacterial sepsis, in COVID-19 sepsis, a higher procalcitonin value is associated with higher SOFA scores (Spearman’ rho coefficient = 0.58 (95% CI: 0.46–0.68), *p* < 0.001) (Figure 3B).

## 3. Discussion

Our study describes a comparative analysis of the hematological changes induced by two different types of pulmonary sepsis in 262 patients admitted to ICU. We demonstrated that some hematological parameters (the absolute count of white blood cells, neutrophils, monocytes) have good discriminative ability between viral and bacterial sepsis, while the best biomarker differentiating these two entities is procalcitonin. Suprisingly, RDW% also had a very good power in identifing patients with bacterial sepsis. Derived hematological indices did not perform well compared with other hematological parameters and have low sensitivity and specificity. From our knowledge, this is the first study to test in an extensive manner the discriminative ability of these parameters between COVID-19 sepsis and bacterial sepsis secondary to bacterial pneumonia.

In the study presented herein, patients with bacterial sepsis were older compared with COVID-19 patients. Severe bacterial pneumonia and subsequently, sepsis, appear more often at the extremes of age, in patients living in nursing homes or with higher degrees of chronic disease severity [26]. In our cohort, 27.4% of patients presented with HCAP and overall, patients with bacterial sepsis had significantly higher Charlson Comorbidity Index (CCI) value. One should take into account the age variable in the CCI calculation and interpret cautiously this difference given the older population in the bacterial sepsis group.

Our results regarding the hematological alterations are in line with data reported by Moser et al., except for lymphocytes [4]. In their study population, patients with bacterial sepsis secondary to pneumonia had a significantly higher proportion of neutrophils (*p* = 0.025), but lower lymphocytes (*p* = 0.033) and platelet numbers (*p* < 0.001). Moreover, procalcitonin (*p* < 0.001) and IL-6 (*p* = 0.036) were higher in the bacterial sepsis group. Lastly, higher eosinophil counts are found in bacterial sepsis patients compared with COVID-19 sepsis, but no difference was observed for the basophil count [27].

Wu et al., specifically compared the characteristics of patients with COVID-19 sepsis and patients with bacterial sepsis secondary to carbapenem-resistant *Klebsiella pneumoniae* (CrKp) pneumonia [28]. Quantitative alterations of the hematological parameters are similar with the ones reported by this study. Patients with CrKp had significantly higher median values for leukocytes, neutrophils, monocytes, NLR and procalcitonin (*p* < 0.05). Furthermore, for the median value of lymphocytes and PLR, the same observation as in our analysis was made. Subjects with CrKp sepsis had lower median PLR values (*p* < 0.05), while COVID-19 sepsis subjects had significantly lower lymphocytes values (*p* < 0.05) [28].

Hematological parameters with good discriminative ability were leukocytes, monocytes and neutrophils with AUCs between 0.76 and 0.78 (*p* < 0.001). Given that both types of sepsis are associated with leukocytosis and neutrophilia, the cut-off values of >16 × 10^3^/mm^3^ for WBC and >14.1 × 10^3^/mm^3^, although seem high, they differentiate between two types of distinctive inflammatory response [4,12]. The cut-off value of >0.69 × 10^3^/mm^3^ for monocytes, even with a value found in the normal range, is explained by monocytosis’ higher frequency in the bacterial sepsis group, while monopenia was observed more often in the COVID-19 group. Quantitative alterations are mostly non-specific, but are used on a daily basis for the assessment of patients with sepsis. From a pathophysiological point of view, they may seem less important compared with the qualitative alterations induced by these two conditions [2,4,5]. On the other hand, laboratory tests studying the qualitative alterations are not widely used, nor widely available. Thus, the identified cut-off values we reported, bring more clinical insight into the differential diagnosis of bacterial and viral sepsis.

Furthermore, Perschinka et al. found that procalcitonin, IL-6 and C-reactive protein were significantly higher in patients with bacterial sepsis, even when compared between bacterial pneumonia and COVID-19 pneumonia [29]. Their results showed that two distinct phenotypes were present and steroid therapy did not represent a confounder in their analysis. This was because these differences were maintained between bacterial and COVID-19 sepsis in patients regardless of steroid treatment. Moreover, in their opinion, the high lactate values seen in bacterial sepsis made the diagnosis of sepsis in COVID-19 infection questionable [29]. The reduced degree of organ involvment, other than the lung, is still debated and is considered by others a solid argument to consider COVID-19 a single-organ disease [30]. These discrepancies can be explained, in part, by the different mechanisms through which the systemic responses are generated and propagated during disease evolution [2,5,31,32,33,34]. Bacteria are capable of producing higher levels PAMPs (pathogen-associated molecular patterns) causing direct injury in different organs. This, in turn, leads to increased production of DAMPs (damage-associated molecular patterns) which will further maintain the inflammatory response and lead to organ failure [33,34]. On the other hand, SARS-CoV-2 primarly manifests with alveolar thrombosis and pulmonary involvement. After the virus enters the blood, there is evidence of organ involvement (other than the lungs), but to a milder extent in terms of organ dysfunction [32,33,35,36,37]. As seen in our study, patients with viral sepsis had lower SOFA scores, mainly based on the high value of the respiratory subscore. This observation was made in a previous study and raised questions regarding the utility of SOFA score in COVID-19 diagnosis, risk stratification and prognosis. This led to the development of a new score by our research group, the COVID-SOFA score, powered to predict 28-day all-cause mortality. The score had significantly higher discriminative ability when compared with the SOFA score alone [38].

Although not specific, RDW%, was found to be elevated in bacterial sepsis through changes into rheological characteristics of the erythrocytes [39]. These alterations are related to disturbances in the structure and function of the red blood cell (RBC) membrane and cytosol [39,40,41,42]. Thus, these will lead to increased RBC sphericity and abnormal deformability. The final result is increased RBC anisocytosis [39,41]. Lastly, the bacterial insult leads to increased release of erythroblasts into circulation [42]. Increased RDW% was found to be associated with mortality risk in COVID-19 patients [43,44] and progression to septic shock in patients with COVID-19 with an AUC of 0.77 [43]. Results from these studies are difficult to be compared with ours because patients were classified as high or normal RDW%. The high RDW group was considered at a value >14.6% [44]. In our study, the cut-off value for RDW as a discriminant between bacterial and COVID-19 sepsis was >14.8%. This value is higher than the upper limit of normality, suggesting that bacterial sepsis has direct and indirect effects on RBC morphology more than COVID-19 sepsis. Thus, we consider RDW% a valuable tool in the differential diagnosis of bacterial sepsis and also, an indicator of disease severity and prognosis. Caution should be taken when using RDW as a discriminant, given that its value can be increased in different other conditions and is dependent on RBC transfusion [45,46,47].

Finally, the model for procalcitonin had an excelent discriminative power, with an AUC of 0.92 (95% CI: 0.87–0.95). In our study, the value of >1.49 ng/mL (95% CI: 1.28–1.9) differentiates between bacterial and COVID-19 sepsis, with a +LR = 13.22 (95% CI: 6.7–21.1). Procalcitonin is considered to have good accuracy for the differentiation of viral and typical bacterial pneumonia with an AUC of 0.79 (95% CI: 0.75–0.82) [48]. For a threshold of 0.1 ng/mL, procalcitonin’s sensitivity was 80.9% (95% CI: 75.3–85.7) and specificity was 51.6% (95% CI: 46.6–56.5). However, in the aforementioned study, not only critically-ill, but all hospitalized patients with pneumoia were inluded [48]. Different thresholds were proposed for the discrimination between: (i) bacterial co-infection between COVID-19 patients at ICU admission [23,49], (ii) bacterial co-infection during hospitalization [50], and (iii) antibiotic stewardship [22,50]. In COVID-19 patients, Atallah et al. reported cut-off values for procalcitonin of >0.25 ng/mL (for positive blood cultures at day 1) and >0.5 ng/mL (for positive sputum cultures at day 1) [49]. The same cut-off values for procalcitonin were reported by May et al. regarding the identification of community-associated infections of COVID-19 patients [23]. Procalcitonin’s sensitivity and specificity reported in these studies vary considerably and are different from the ones we reported.

Although observed in both types of sepsis, the stronger correlation between procalcitonin and disease severity in the viral sepsis group was an interesting finding of our study. In COVID-19 patients, higher procalcitonin levels are correlated with the degree of organ damage and subsequently, disease severity [51,52]. Moreover, higher procalcitonin values predict the development of secondary infections during ICU stay [49,50]. Lastly RDW% values were also associated with disease severity in bacterial sepsis group. Our observations are similar with other reports for both RDW% and procalcitonin [53,54].

The cut-off value for procalcitonin in our study was higher than the ones reported in the aforementioned studies. Given that increased procalcitonin levels were associated with disease severity in COVID-19, we suggest that procalcitonin should be used with caution. Higher procalcitonin values should not exclusively be considered secondary to bacterial pneumonia, unless viral etiologies were excluded.

Our study has some limitations. Firstly, the retrospective nature of this manuscript prones our results to selection bias. Secondly, only 262 patients from a single center were included in this study, henceforth our findings can not be extensively applied to different types of population, increasing the risk of geographic bias. Thirdly, C-reactive protein measurement was not available in the bacterial sepsis group, nor was measurement of cytokines such as IL-1, IL-6 or TNF-α as these biomarkers are extensively studied in sepsis, regardless of etiology. Furthermore, although patients with bacterial sepsis presented with different types of pneumonia (CAP, HCAP, VAP), in the viral sepsis group, only patients with COVID-19 sepsis were analyzed. This limits the extension of our results to all types of viral sepsis. Moreover, patients with COVID-19 and bacterial co-infection were not introduced in this study.

## 4. Materials and Methods

### 4.1. Study Population

We conducted a retrospective, observational and comparative analysis on a cohort of 262 patients with sepsis secondary to bacterial pneumonia and viral sepsis secondary to COVID-19, admitted to the intensive care unit of a tertiary center (Elias Univeristy Emergency Hospital of Bucharest). Two study periods were chosen: (i) patients admitted with sepsis secondary to bacterial pneumonia, during a period of 24 months (January 2017–December 2018), (2) patients admitted with viral sepsis due to COVID-19 during a period of 16 months (August 2020–December 2021). Local ethics committee of Elias University Emergency Hospital approved this study. Sepsis was defined according to Sepsis-3 criteria [1]. The inclusion criteria for patients with bacterial sepsis were age >18 years, bacterial pneumonia confirmed based on clinical findings and chest X-ray or CT scan and (i) positive bacterial cultures (qualitative or quantitative assessment) or (ii) without an identified pathogen but with clinical response to antibiotic therapy. Exclusion criteria were patients with confirmed viral pneumonia and bacterial co-infection and patients admitted in ICU initially with sepsis secondary to an infection other than pneumonia. Inclusion criteria for patients with viral sepsis were age > 18 years and COVID-19 confirmed through real-time polymerase chain reaction (RT-PCR) and chest X-ray or CT scan. The exclusion criteria for patients with viral sepsis were bacterial co-infection (positive qualitative or quantitative bacterial cultures regardless of site, clinical or radiological findings suggestive of bacterial co-infection), patients admitted in our ICU but mechanically ventilated for > 48 h in a different ICU. In both groups, patients with ongoing radio-, chemo- or immunotherapy, patients with end-stage organ disease (cardiac, kidney, lung, liver), patients with end-stage cancer or severe hematological diseases, patients with missing data and those not meeting Sepsis-3 criteria were excluded.

### 4.2. Data Collection and Analysis

The following data were collected: demographic (age, gender), associated diseases (cardiac, respiratory, kidney, liver, diabetes mellitus, obesity) and Charlson Comorbidity Index values. Moreover, the following data were collected at the moment of sepsis diagnosis: mechanical ventilation and vasopressor drugs requirement, hematological parameters (absolute count of white blood cells, neutrophils, lymphocytes, monocytes, platelets and immature granulocytes, red blood cell distribution width (RDW%) and platelet distribution width (PDW%)), P/F ratio, procalcitonin values and SOFA (Sequential Organ Failure Assessment) score.

Data was studied depending on the type of sepsis: bacterial and viral. Results were reported for the whole sample and for both groups separately. Also, based on the hemogram parameters, five derived indices were calculated: (1) neutrophil-to-lymphocyte ratio (NLR), (2) monocyte-to-lymphocyte ratio, (3) systemic inflammatory index (SII, (neutrophil × platelet)/lymphocyte ratio), (4) platelets-to-lymphocyte ratio and (5) derived neutrophil-to-lymphocyte ratio (dNLR, absolute neutrophil count/(white blood cells—absolute neutrophil count)).

### 4.3. Statistical Analysis

Firstly, data was tested for normality of distribution using the Kolmogorov-Smirnov test. Categorical variables were expressed as absolute (number) and relative (percentage) frequency. After crosstabulation, categorical data were compared based on the Chi-square test. Continous data was expressed as median and interquartile range [IQR: Q1–Q3]. The Mann-Whitney U test was used to asses the differences between continous and not normally distributed data across two independent groups. Also, bivariate analysis using Spearman’s rho coefficient was performed in order to study the rang correlation between two continous variables. The results from the bivariate analysis were plotted as scatter diagrams, including trend lines with a LOESS smoothing span of 99%. Receiver Operating Characteristics (ROC) analysis was performed to test the discriminative ability of hematological parameters and procalcitonin between bacterial and viral sepsis. For every variable, a ROC curve was plotted and the area under the curve (AUC) was calculated. The cut-off value was identified based on the Youden index. Sensitivity (Sn%), specificity (Sp%), positive likelihood ratio (+LR) and negative likelihood ratio (–LR) together with 95% confidence intervals (95% CI) were calculated for the identified cut-off value. The 95% CI were also reported for the AUC, Youden index, cut-off values and Spearman’s rho coefficient of correlation. ROC curves were compared using the DeLong method [55]. The level of significance was established at an alpha level <0.05.

The statistical analysis was conducted using IBM Statistical Package for Social Sciences (SPSS) for Windows^®^ version 20.0 (IBM Corporation, Armonk, NY, USA) and MedCalc for Windows, version 20.106 (MedCalc Software^®^, Ostend, Belgium).

## 5. Conclusions

In conclusion, bacterial and viral sepsis secondary to bacterial pneumonia and COVID-19, respectively, have different hematological patterns, as it was demonstrated by the good discriminative ability of leukocytes, neutrophils, monocytes, PLR and eosinophils. Other hematological parameters were fair (MLR, platelets, basophils, lymphocytes and NLR) or no (dNLR, SII) discriminants at all. Moreover, the particular involvement of other organs was once again outlined in this study since patients with bacterial sepsis presented higher degrees of non-respiratory organs dysfunction, while in COVID-19 sepsis, the mainstay of organ involvement was the severe acute respiratory failure. RDW% is a very good discriminant between bacterial and COVID-19 sepsis, reiterating the effects of bacterial sepsis on red blood cells. Lastly, the best discriminant between them remains procalcitonin, but one should keep in mind that a definitive threshold is not set and that in both types of sepsis, procalcitonin level is strongly correlated with disease severity.

## Figures and Tables

**Table 1:** Baseline Characteristics of the Study Population.

*n* (%) or Median [IQR: Q1–Q3]	Total Sample *n* = 262	Bacterial Sepsis *n* = 124	Viral Sepsis *n* = 138	*p* Value
Age	68 [58–77]	73 [66–81]	64 [53–72]	<0.001 *
Gender (male)	158 (60.3%)	74 (59.7%)	84 (60.9%)	0.844 *
Obesity	158 (60.3%)	67 (54%)	91 (66%)	0.062 *
Cardiac disease	195 (74.4%)	77.4% (96)	71.7% (99)	0.293 *
Respiratory disease	32 (12.2%)	20 (16.1%)	12 (8.7%)	0.067 *
Chronic kidney disease	41 (15.7%)	31 (25.2%)	10 (7.2%)	<0.001 *
Diabetes mellitus	104 (39.8%)	50 (40.7%)	54 (39.1%)	0.802 *
Hepatic disease	23 (8.8%)	15 (12%)	8 (5.8%)	0.113 *
Charlson comorbidity index	5 [3–7]	7 [5.3–9]	4 [2–5]	<0.001 **
Hemoglobin (g/dL)	11.8 [10.18–13.6]	10.5 [9.1–12.3]	12.8 [11.3–14.1]	<0.001 **
RDW%	14.6 [13.3–15.8]	15.7 [15–17.2]	13.6 [12.8–14.5]	<0.001 **
Platelets × 10^3^/mm^3^	226 [169–295]	188 [127–260]	253 [193–316]	<0.001 **
PDW%	12.9 [11.7–14.7]	13.1 [11.8–15]	12.8 [11.4–14.5]	0.215 **
White blood cells × 10^3^/mm^3^	13.11 [9.14–18.91]	17.7 [12.7–23]	10.2 [7.85–13.8]	<0.001 **
Neutrophils × 10^3^/mm^3^	11.48 [7.55–16.49]	15.5 [10.7–20.2]	8.9 [6.6–12.4]	<0.001 **
Lymphocytes × 10^3^/mm^3^	0.8 [0.54–1.17]	0.95 [0.58–1.4]	0.74 [0.52–1.06]	0.005 **
Monocytes × 10^3^/mm^3^	0.59 [0.33–0.87]	0.82 [0.56–1.16]	0.45 [0.28–0.66]	<0.001 **
Eosinophils × 10^3^/mm^3^	0.001 [0.00–0.02]	0.01 [0.001–0.07]	0.001 [0.00–0.001]	<0.001 **
Basophils × 10^3^/mm^3^	0.02 [0.01–0.03]	0.02 [0.01–0.038]	0.01 [0.01–0.02]	<0.001 **
NLR	14.07 [8.75–21.41]	15.6 [9.4–26.7]	12.2 [8.4–19.3]	0.01 **
dNLR	7.25 [5.22–11]	7.3 [4.5–10.5]	7.2 [5.7–11.6]	0.584 **
MLR	0.64 [0.42–1.16]	0.92 [0.5–1.4]	0.55 [0.39–0.8]	<0.001 **
PLR	268 [166–433]	213 [122–311]	350.4 [227–471]	<0.001 **
SII	2871 [1575–5070]	2770 [1348–5151]	2991 [2012–5039]	0.314 **
iGr × 10^3^/mm^3^	0.11 [0.06–0.34]	0.15 [0.06–0.44]	0.1 [0.05–0.23]	0.023 **
Procalcitonin ng/mL	0.8 [0.22–5]	5.5 [1.6–23.2]	0.25 [0.13–0.6]	<0.001 **
Mechanical ventilation	192 (73.3%)	89 (71.8%)	103 (74.6%)	0.601 *
P/F ratio	142 [89–230]	214 [155–321]	100 [75–133]	<0.001 **
Vasopressor use	90 (34.4%)	76 (61.3%)	14 (10.1%)	<0.001 *
SOFA score	5 [4–9]	8 [6–12]	4 [3–5]	<0.001 **

* Chi-square Test, ** Mann-Whitney U Test; RDW% = red blood cell distribution width, PDW% = platelet distribution width, NLR = neutrophil-to-lymphocyte ratio, dNLR = derived neutrophil-to-lymphocyte ratio, MLR = monocyte-to-lymphocyte ratio, PLR = platelet-to-lymphocyte ratio, SII = systemic inflammatory index, iGr = immature granulocytes.

**Table 2:** Discriminative Analysis Between Bacterial and Viral Sepsis.

	AUC 95% CI	*p* Value	Youden Index 95% CI	Cut–Off 95% CI	Sn% 95% CI	Sp% 95% CI	+LR 95% CI	–LR 95% CI
Procalcitonin	0.92 0.87–0.95	<0.001	0.71 0.61–0.77	>1.49 1.28–1.9	76.6% 68.2–83.7	94.2 88.9–97.5	13.22 6.7–21.1	0.25 0.18–0.35
RDW%	0.87 0.82–0.91	<0.001	0.66 0.57–0.75	>14.8 14.6–15.2	80.7% 72.6–87.2	85.5% 78.5–90.9	5.56 3.68–8.42	0.23 0.16–0.33
Leukocytes	0.78 0.72–0.83	<0.001	0.5 0.4–0.58	>16 14.4–17.3	64.5% 55.4–72.9	85.5% 78.5–90.9	4.45 2.91–6.81	0.41 0.32–0.53
Monocytes	0.77 0.71–0.82	<0.001	0.44 0.32–0.52	>0.69 0.55–0.9	63.2% 53.6–72	81.2% 73.6–87.3	3.35 2.31–4.87	0.45 0.35–0.58
Neutrophils	0.76 0.7–0.82	<0.001	0.49 0.37–0.57	>14.1 10.9–14.9	64.5% 55.4–72.9	84.1% 76.9–89.7	4.05 2.7–6.07	0.42 0.33–0.54
Eosinophils	0.72 0.6–0.7	<0.001	0.43 0.32–0.54	>0.001 0.00–0.001	66.1% 57.1–74.4	76.8% 68.9–83.6	2.85 2.1–4	0.44 0.34–0.57
PLR	0.71 0.64–0.76	<0.001	0.35 0.23–0.43	≤259 226–392	65.3% 56.3–73.6	69.6% 61.2–77.1	2.15 1.62–2.85	0.5 0.38–0.65
MLR	0.69 0.61–0.75	<0.001	0.34 0.19–0.4	>0.73 0.59–1.17	61% 51.8–69.6	72.9% 64.3–80.3	2.25 1.64–3.08	0.54 0.42–0.68
Basophils	0.68 0.6–0.72	<0.001	0.27 0.17–0.39	>0.01 0.001–0.02	67.7% 58.8–75.9	59.4% 50.7–67.7	1.67 1.32–2.11	0.54 0.41–0.73
Platelets	0.67 0.6–0.73	<0.001	0.28 0.16–0.36	≤189 169–281	51.6% 42.5–60.7	76.8% 68.9–83.6	2.23 1.57–3.15	0.63 0.51–0.77
Lymphocytes	0.6 0.53–0.7	0.005	0.21 0.09–0.3	>0.85 0.33–1.04	58.1% 48.9–66.9	63% 54.4–71.1	1.57 1.21–2.05	0.67 0.52–0.85
NLR	0.59 0.52–0.66	0.01	0.18 0.09–0.25	>27.24 24.5–38.4	25% 17.7–33.6	93.5% 88–97	3.83 1.9–7.73	0.8 0.72–0.9
iGr	0.58 0.52–0.67	0.023	0.17 0.08–0.24	>0.14 0.01–0.41	50.8% 41.7–59.9	66% 57.4–73.8	1.49 1.12–1.99	0.75 0.6–0.93
SII	0.54 0.46–0.6	0.32	–	–	–	–	–	–
dNLR	0.52 0.47–0.58	0.59	–	–	–	–	–	–

AUC = area under the curve, 95% CI = 95% confidence interval, Sn% = sensitivity, Sp% = specificity, +LR = positive likelihood ratio, –LR = negative likelihood ratio, RDW% = red blood cell distribution width, NLR = neutrophil-to-lymphocyte ratio, dNLR = derived neutrophil-to-lymphocyte ratio, MLR = monocyte-to-lymphocyte ratio, PLR = platelet-to-lymphocyte ratio, iGr = immature granulocytes, SII = systemic inflammatory index.

## Data Availability

The data presented in this study are available on reasonable requestfrom the corresponding author.
